# Metabolic disharmony and sibling conflict mediated by T6SS

**DOI:** 10.15698/mic2018.05.632

**Published:** 2018-04-04

**Authors:** Vera Troselj, Daniel Wall

**Affiliations:** 1 Department of Molecular Biology, University of Wyoming, Laramie, Wyoming.

**Keywords:** physiological heterogeneity, myxobacteria, type VI secretion system, cooperation, conflict

## Abstract

Bacteria in nature live in taxonomically complex communities where multitude of species and strains inhabit the same niches and compete for limited resources and space. Surviving in these competitive environments requires mechanisms to recognize and associate with kin and to discriminate against non-kin to increase reproductive success among close relatives. Some of the mechanisms bacteria use to address genetic differences are surface receptors, diffusible signals (e.g. quorum sensing) and toxin-immunity systems (e.g. type VI secretion system (T6SS)). Another way individuals vary within bacterial populations is their physiological states. This means that among clonal cells there is cell-to-cell variability in cells’ proteome, growth rates, age and cell damage loads caused by stochastic differences in gene expression/metabolism and variations in microenvironmental stimuli. While physiological heterogeneity benefits some bacteria by allowing populations to bet-hedge their survival odds in changing environments by expressing different phenotypes, it can also be harmful in cases where fitness depends on coordinated behaviors and synchronized actions by many cells; a function of particular importance to social bacteria. *Myxococcus xanthus* is a non-pathogenic soil bacterium known for its complex social and coordinated behaviors such as swarming, predation and formation of spore-filled fruiting bodies. These behaviors depend on *M. xanthus* ability to synchronize the actions of many cells within a population. Considering the collective nature of *M. xanthus*, we asked how do physiological differences affect cell-cell interactions in this species*. *To address this question, we investigated the interactions between two genetically related but physiologically distinct populations. We found that *M. xanthus* uses T6SS to eliminate less fit cells from their population and identified toxic effector and cognate immunity protein (TsxEI) that mediates this sibling antagonism.

 Our approach involved creating amino acid auxotrophs and mixing them with their parent prototroph on minimal media where the auxotroph starves while the prototroph grows, thus creating conditional physiological differences between these populations. We predicted one of three possible outcomes; neutral, cooperative (e.g. auxotroph benefits from prototroph by cross-feeding) or antagonistic interactions. As a control, the auxotroph and prototroph strains were mixed under conditions where the populations were physiologically similar - on rich media where both grew or on starvation agar where both starved. Importantly, on minimal media the starving auxotrophs were killed by the growing prototrophs. In contrast, when the two populations were physiologically similar (rich and starvation agar) there was no antagonism. Furthermore, this antagonism depended on gliding motility.

To investigate the mechanism that mediates sibling killing, we screened *M. xanthus* strains with defects in known or putative toxin systems. We found that sibling antagonism was abolished when the auxotroph was mixed with a prototroph that contained a mutation in the T6SS. T6SS is a contractile nanomachine that injects toxic effectors into neighboring cells. T6SS is best known as a weapon used in bacterial warfare, although it is also used as a virulence and self/nonself-recognition determinant. T6SS effectors are delivered through a contractile tube made out of Hcp protein hexamers with a tip composed of VgrG trimers and PAAR domain containing proteins. Along with being structural components of T6SS, VgrG and PAAR domain containing proteins can have additional functions where they are fused with effectors or act as adapter proteins for effector delivery. *M. xanthus* has all of the core T6SS genes within one operon, and an additional orphan *vgrG* gene. While T6SS role in inter-species and inter-strain competition is well established, our report is the first where T6SS is used against sibling cells; albeit siblings with physiological differences. We further showed that both *vgrG* genes were required for antagonism, suggesting the two VgrG proteins play non-redundant roles.

In search of a toxin effector, we used bioinformatics to identify two adjacent candidate effector-immunity genes (*tsxEI*). TsxE orthologs are also found in related myxobacterial species. By genetic analysis we found that a *tsxE* mutant was incapable of killing an auxotroph and that killing was restored by ectopic expression of TsxE. Moreover, a *tsxEI* deletion mutant was killed on rich media by its parent strain, but not by a T6SS mutant. Consistent with our earlier findings with prototroph/auxotroph antagonism, killing of the TsxEI mutant by the parent strain was blocked on starvation agar or when the competitor strain was nonmotile. We conclude that TsxE is a T6SS effector employed by *M. xanthus* against less fit (starving) siblings and that TsxI is the cognate immunity factor. We then asked what made auxotrophs susceptible to killing by their prototroph siblings, considering that both populations contain T6SS and TsxEI. To address this key question we investigated the relative levels of T6SS proteins in starving cells compared to growing cells. In this regard we found that the relative levels of TsxI and Hcp decreased in starving cells. Conversely, in growing cells, the levels of both proteins actually increased over time.

Based on these results we propose a model where starvation causes T6SS and TsxEI cellular levels to be depleted. In turn this makes starving *M. xanthus* cells susceptible to T6SS attack by growing cells (Figure 1). This sibling antagonism depends on motility and requires both VgrG proteins. VgrG1 is encoded within the T6SS operon and as such may play a critical role in T6SS structure, while VgrG2, which is encoded immediately upstream to *tsxEI*, might act as a specific adapter for TsxE. Considering that motility plays many crucial roles in *M. xanthus *social behaviors, e.g. swarming, predation, outer membrane exchange and development, its requirement for T6SS function provides yet another example where motility is coupled to social behaviors in *M. xanthus*.

**Figure 1:**
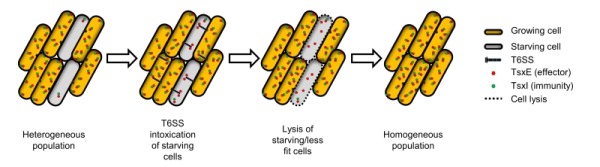
FIGURE 1: Model for how* M. xanthus* eliminates starving/less fit cells from a physiologically heterogeneous population through T6SS attack. *M. xanthus* population is initially depicted as a mixture of growing and starving cells. Starving cells have lower levels of TsxEI and therefore are susceptible to TsxE intoxication. T6SS is expressed at elevated levels in growing cells and TsxE is delivered to neighboring siblings. As a consequence, starving cells are intoxicated, lysed, and removed from the population. This results in increased physiological homogeneity and improved fitness of the population to conduct coordinated multicellular functions.

One possible role for this sibling antagonism is to eliminate less fit cells from populations. In this scenario, *M. xanthus* populations consist of heterogeneous phenotypes, where some cells have lower fitness (e.g. starvation) and, consequently, their levels of TsxI are decreased. In turn these cells are eliminated by their siblings by T6SS-mediated attack (Figure 1). This culling of individuals increases the overall fitness of the population by making cells more homogenous, thus facilitating their ability to coordinate and synchronize social behaviors. The lysis of less fit cells also releases nutrients that may help the other cells grow. Furthermore, this mechanism can impact *M. xanthus* decision to undergo multicellular development and sporulate or to continue vegetative growth. Sporulation is a committed and energetically costly developmental program and as such is undertaken only when a consensus is established within a population. We hypothesize that TsxEI mediated antagonism delays this decision by eliminating cells that enter developmental process prematurely and, as suggested above, by cannibalizing nutrients released by lysed clonemates.

One of the interesting questions that remains to be answered is what signaling pathway(s) regulates T6SS and TsxEI levels in starving cells? In other words, is this regulation a general response to nutrient depletion or a specific response to amino acid starvation, e.g. stringent response? For example, would a depletion of a different nutrient, such as nucleotide pool, result in the same T6SS response? As a predatory bacterium, *M. xanthus* relies on protein as the primary carbon and energy source and thus its physiology is fine tuned to respond to amino acid starvation by initiating a stringent response and consequently changing its proteome. However, our findings to date do not support a simple stringent response as governing sibling antagonism. Other remaining questions include what is the mechanism and target for TsxE toxicity, and does TsxEI have another role outside of the sibling antagonism described here? It is also likely that *M. xanthus*, similar to other bacterial species with this secretion system, uses T6SS for inter-species and inter-strain bacterial warfare, possibly by employing different (and less conserved) types of toxic effectors.

